# Comparing oil based ointment versus standard practice for the treatment of moderate burns in Greece: a trial based cost effectiveness evaluation

**DOI:** 10.1186/1472-6882-11-122

**Published:** 2011-12-01

**Authors:** Vilelmine J Carayanni, Evangelia G Tsati, Georgia C H Spyropoulou, Fotini N Antonopoulou, John D Ioannovich

**Affiliations:** 1 Department of Public Health, Faculty of Health and Caring Professions Technological Educational Institute of Athens, Saint Spyridonos Street, Egaleo 2210, Greece; 2 5 Haras St., Halandri 1523, Athens, Greece; 3 Department of Plastic Surgery, Thriasion Hospital, 19018 G. Gennimata Avenue, Magoula Attiki, Greece; 4 "G. Gennimatas" General State Hospital of Athens, 154 Mesogion Avenue, Greece; 5 Author deceased. In memory of Professor J. Ioannovich †(2003), Department of Plastic Surgery, Microsurgery and Burn Center, "G.Gennimatas" General State Hospital of Athens, Athens, 154 Mesogion Avenue, Greece

## Abstract

**Background:**

The local treatment of burn wounds has long been a subject of debate. The objective of this study was to compare the cost and the effectiveness of Moist Exposed Burn Ointment -MEBO versus a combination of *povidone iodine *plus *bepanthenol *cream for partial thickness burns.

**Methods:**

The study was carried out in the Burn Center of a state hospital in Athens, Greece. 211 patients needing conservative therapy were prospectively selected according to the depth of the burn wound. The treatment was allocated according to the Stratified Randomization Design. The outcomes measured were mean cost of in-hospital stay, rate of complications, time of 50% wound healing, pain scores, in hospital stay diminution. We have adopted a societal perspective.

**Results:**

In the total groups MEBO presented lower cost, (although not significantly different: p = 0.10) and better effectiveness. The data suggest that MEBO is the dominant therapy for superficial partial burn wound with significantly lower costs and significantly higher effectiveness due to a lesser time of recovery and consequently lower time of hospitalization and follow-up. MEBO presented similar percentages of complications with the comparator, lower pain levels and smaller time of no healthy appearance of the burn limits for superficial partial thickness burns.

**Conclusions:**

The data suggested that topical application of MEBO may be considered for further investigation as a potential first-line treatment modality for superficial partial thickness burns.

**Trial registration:**

The trial has been registered on the International Standard Randomised Controlled Trial Number Register (ISRCTN) and given the registration number ISRCTN74058791.

## Background

The local treatment of burn wounds has long been a subject of debate. Several agents in many forms (creams, dressings, gauzes etc.) have been applied to improve and accelerate the healing process [1]. Management of the burn wounds with natural products like herbs (tea tree, aloe vera, shea butter), or animal products (honey, emu oil) is also a worldwide common practice.

Moist exposed burn ointment (MEBO) is an oil-based ointment that contains sesame oil, beta-sitosterol, berberine, and other small quantities of plant ingredients [2] developed at the China National Science and Technology Centre in Beijing in 1989 [3]. The main ingredient of MEBO is beta-sitosterol, which has been shown to have anti-inflammatory effects, [4] and berberine, which has antimicrobial effects [4]. Laboratory tests have indicated that MEBO was not a mucocutaneous irritant nor was it orally toxic to rats; repeated cutaneous patch tests in humans did not show any potential for dermal irritation or sensitization [5]. Clinical and experimental investigations have shown that MEBO has analgesic and antimicrobial effects, and reduces water evaporation from the burn surface [6-12]. Clinical studies have found that MEBO promotes debridement, epithelial repair and is associated with better scar quality [6-17]. In three consecutively conducted clinical studies, Atiyeh *et al.*, found that the MEBO exhibited a beneficial prophylactic effect on primary and secondary wound healing with scar quality superior in wounds treated with MEBO [10.11.15]. In a prospective comparative study comparing healing with MEBO to conventional occlusive dressings, the biologic healing with MEBO, as determined by trans-epidermal water loss (TEWL) measurements, occurred at an extremely significant earlier stage and was associated with better scar quality [16].

Although there is an important number of a considerable study in the Chinese language [18-26] regarding the safety and the effectiveness of MEBO, few are the studies evaluating this alternative therapy worldwide. It is pointed out that there is a poor record of Trial Based Economic Evaluations comparing MEBO [5-7]. Information for these studies is given on Table 1.

**Table 1:** Principal characteristics of Trial Based Economic Evaluations comparing MEBO.

Author and country	Patient group	Study type, setting and perspective	Comparators	Main results
1) Ang et al, 2001 Singapore	115 (started) patients (6-80 years) with partial thickness (TBSA: < 40%) burns to the face excluding chemical and electrical burns.	Cost consequences, single centre study, secondary care. Perspective adopted: health care system	Silver sulfadiazine cream (C)	The median time to 75% healing was 17.0 and 20.0 days in MEBO and conventional therapy groups, respectively (Hazard Ratio: HR: 0.67; 95% CI: 0.41-1.11; p = 0.11), (similar efficacy). Bacterial infection rates were similar between the two groups (HR: 1.10; 95% CI: 0.59-2.03; p = 0.76). MEBO imparted a greater analgesic effect in the first 5 days of therapy and reduced hospital costs by 8%
2) Atiyeh B. S. et al, Egypt, 2002	40 patients between (5-54 years) with superficial partial thickness burns 5-20% TBSA in adults and 5-15% TBSA in children, excluding chemical and electrical burns and patients with visual, mental or physical disabilities pregnant or lactating women as females at pregnancy risk.	Cost Benefit under a clinical prospective multi-center (five centers) study, secondary care. Perspective adopted: health care system	Silver sulphadiazine, Sofratulle Chlorhexidinetulle, Nitrofurazone, Quadriderm (betamethasone + chlorocresol+clioquinol +gentamicin+tolnaftate), Dexpanthenol, NitrofurazoneSavlon (cetrimide + chlorhexidine) Hydrogen peroxide Povidone-iodine	Patients not treated with MEBO application required statistically significant (p < 0.01) longer hospitalization. (30%) The time spent by nurses (p < 0.01) and doctors (p < 0.05), were significantly lower in MEBO as well as overall direct costs (p < 0.01.)
3) Atiyeh B. S. et al., 2004, Saudi Arabia	52 (started) patients (2-58 years) with a second degree TBSA burns of 5 to 35% burn (> 15% TBSA for children and > 20% for adults), excluding chemical and electrical burns	Cost consequences under a clinical prospective multi-center (14 centers) study, secondary care. Perspective adopted: health care system	Silver Sulfadiazine, Extract cepae 10%, heparin sodium 5000 iu and allantion, Panothenic acid, Chlorohexidine, Fucidic acid Bacitracin zinc and neomycin sulphate, Povidone iodine, Sofratulle.	Significant differences in favour of MEBO group concerning the reduction of 20.24% in hospitalization time (p = 0.0056), the total hospitalization cost (p = 0.025), the total time spent by physicians and nurses, the analgesic cost per day reduced by 60.8%) per course (p = 0.0135) and 55.88% per day (p = 0.0271). The other differences aren't significant.

## Methods

### Aims of the trial

This study is the first conducted in Greece comparing these therapies. The aim of this trial is to test the hypothesis that MEBO substantially reduces the time of wound healing and is the more cost effective treatment modality for the local treatment of partial thickness burns compared to the standard practice. Specifically, we aimed at testing the hypotheses of treating partial thickness burns with MEBO:

i. Reduces hospitalization time and wound healing [10-17]

ii. Reduces recovery costs [11,12].

iii. Reduces pain levels [10-12]

iv. Does not increase complication rates [10-12]

### Study population

**Figure 1:**
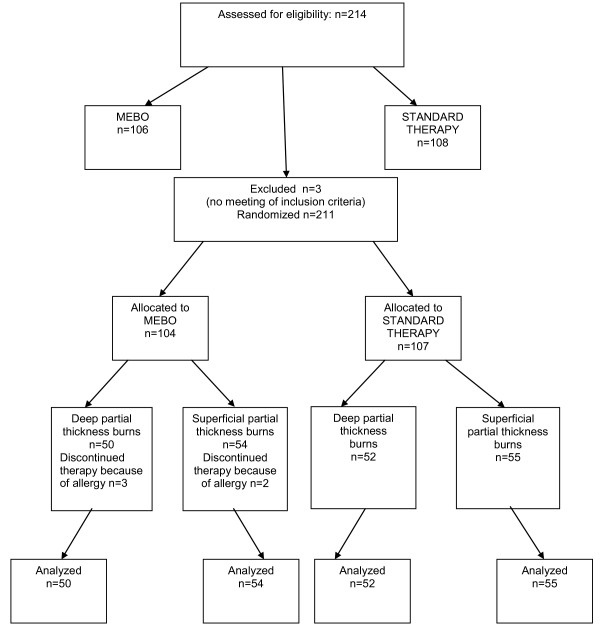
**Participant flow through the trial**.

The patient inclusion criteria were: 1. absence of cancer and diabetes 2. Total Burns Surface Area (TBSA) < 15%. 3. Thermal burns 4. No need of surgical operation 5. Need for hospitalization. 211 (214 randomized) patients, aged between 18-75 years were prospectively selected. Three patients were excluded because of violation of the inclusion criteria (need of surgical operation). The flow of the participants is described in Figure 1.

### Study design and protocol

#### Randomization

The research was carried out in "G.Gennimatas", the Regional General Hospital of Athens, Clinic of Plastic Surgery. The evaluation was carried out under a society perspective. We compared *MEBO *with the combination of *povidone iodine *plus *bepanthenol *cream (standard therapy). The reason for selecting Povidone iodine as comparator was based on the fact that in Greece, up to this day, it is the standard practice of therapy for burns of this kind. The setting of our study used Povidone Iodine solution followed by some moisturizing, healing promoting cream (mainly bepanthenol cream) as a standard burn wound treatment.

We opted for stratified randomization [26] according to the thickness degree to prevent large imbalances. In each therapy group we had 2 sub groups according to the thickness degree:

1) Patients with superficial partial thickness burns who attracted the greatest interest because of an application possibility of the indicator Transepidermal Water Loss as effectiveness indicator. In this category were classified the patients presenting Transepidermal Water Loss < 60 gr/m^2^/h in the first day.

2. Patients with deep partial thickness burns, presenting (Transepidermal Water Loss -TEWL ≥ 60 gr/m^2^/h.

The follow up duration was 18 days.

The procedures followed in the study were in accordance with the recognized ethical standards and with the Helsinki Declaration. The protocol of the study was approved by the Scientific Committee of General Regional Hospital of Athens. The patients provided written consent for participation prerandomization after they were given a full explanation of the treatment options and the manner of treatment allocation. The allocation was carried out by the staff of outpatient reception desk of the Clinic. Patient Envelopes were provided for patients requiring treatment allocation in each group. These were numbered sequentially and a list was provided with the envelopes and completed with the trial number and patient name. The date when the envelope was opened (i.e., the date of randomization) was added. Randomly, alteration was used of permuted 20 sub-blocks of sizes from 1-3 for deep partial thickness burns group and 25 sub-blocs of the same size for the superficial partial burn groups. Blinding the treatments was not possible because Povidone iodine has a characteristic color and odor. Blinding was made only for persons evaluating treatment outcomes in order to eliminate classification bias.

#### Wound care

The local agents- MEBO and Povidone Iodine -, were applied twice per day by the assistance of nursing personnel. Bepanthenol cream was self-applied or by assistance of the nursing personnel. It was applied twice per day after the third or fourth day of therapy with povidone iodine according to the degree of re-epithelialization. The burn wounds (both groups) were also treated and lightly debrided by antiseptic in the shower every second day by the nursing staff, or by medical personnel. Also, dressing of the burn wounds during hospitalization was not applicable primarily because of considerable shortcomings in nursing personnel. That is in the Clinic of Plastic Surgery of the Regional General Hospital of Athens "G. Gennimatas", the open technique was used for the burn wounds and required less medical care and was less expensive in terms of hospitalization cost.

#### Primary clinical outcomes

The measured outcomes were:

1. For all groups of patients the mean reduction- in days- of in-hospital stay (standard of sojourn according to the experts: = 10 days) [27]. That is, the one therapy was more effective than the other, if it allowed for greater gain in hospitalization days than the other therapy did.

2. For patients with superficial partial thickness burns, the time of recovery using the TEWL indicator by considering as recovery the 50% diminution of the TEWL of the first day. The use of 2 different effectiveness measures was justified by the fact that TEWL indicator wasn't valid in the case of deep partial thickness burns. Doctors utilized TEWL indicator measurement habitually on 1^st^, 4^th^, and 7^th ^day and after discharge during the visits.

### Costs

The cost measured was the cost of in-hospital stay including the cost of medicines and of biochemical examinations and, after discharge, the cost per medical visit and cost per medicines. These costs reflected the full economic costs for the National Health Service and the patients. Also, the time of hospitalization constituted the time of incapacity for work with direct social implications. The reference year was 2006. Additionally, the total times spent per course by doctors and nurses were calculated and evaluated independently [28].

The cost is described analytically on Table 2. No discount rate was applied, as the study period for each participant was < 1 year. Other costs such as interviews by phone were excluded because of their low impact on the costs. Other indirect costs such as travel time and waiting time were trivially small and did not differ across regimens and consequently their inclusion would have no important effect on the final results ("rule of reason criterion") [28].

**Table 2:** Costs per patient generated during hospitalisation and after discharge (2006 prices)

Cost type	Health service type	Unit	Quantity	Cost per patient (€)
In hospital costs	Hospitalization	Day	1	73.37
	Time spent by personnel and nurses	Course of treatment	1	No standardized (in minutes)
	Medicine	Sodium Fluoride	1000 ml	0.74 per day*1 day
	Medicine	Povidone iodine scrub	1000 ml	3.65 integral cost price
	Medicine	Tears natural	0.6 ml	0.18 per day
	Medicine	Povidone iodine solution	240 ml	1.24 integral cost price
				
In hospital costs and after discharge	Medicine	MEBO	45 ml	10.00 integral cost proposed price
	Medicine	Bepanthenol cream	100 gr	6.78 integral cost price
	Medicine	Paracetamol	(500 gr)	0.34 integral cost price
	Medicine	Paracetamol plus Codeine	(400 mg+50 mg)	0.31 integral cost price
	Physician's visit	Visit	1	3.00
				
Additional in-hospital costs of events	Laboratory tests	Antibiogramm	1	5.22
	Laboratory tests	Biochemical examinations	1	5.22
	Medicine	Ciprofloraxine, (Vial)	200 mg	2 * €14.49*8 days
	Medicine	Ammoxycillin, (Vial)	1 gr	3* €1.17*8 days
	Medicine	Ammoxyciline+Clavulanic acid (Vial)	600 mg	3* €1.49 *8 days
	Medicine	Clindamycine (Vial)	600 mg	2*8.20 *8 days
	Medicine	Cefotaxim (Vial)	500 mg	2*€1.88 *8 days
	Medicine	Dimethindene (cream)	30 gr	1.53 integral cost price
	Medicine	Methylprednisonole (Vial)	1 gr	7.42 per day

No other measurements of production and productivity losses were made because of no provision of these approaches for taking into account unpaid work, in particular domestic work, or the time of those not in active employment [28]. Such were the cases of retired persons (15 out of 104 in the MEBO group, 16 out of 107 in the standard therapy group) unemployed (1 out of 104, 3 out of 107), housework persons (3 out of 104, 2 out of 107).

### Incremental Cost effectiveness

The formula for the incremental cost effectiveness ratio of the new therapy N versus the existing therapy S (μ_C _and μ_E _being the mean cost and the mean final outcomes effectiveness) is given below:

$$\bar{\bar{ICER}}\;\;=\;\;\frac{\bar{\bar{\mu_{CN}^{}}}\;\;-\;\;\bar{\bar{\mu_{CS}^{}}}}{\bar{\bar{\mu_{EN}^{}}}\;\;-\;\;\bar{\bar{\mu_{ES}^{}}}}\;\;=\;\;\frac{\bar{\bar{\mu_{\Delta C}^{}}}}{\bar{\bar{\mu_{\Delta E}^{}}}}$$

where $\bar{\bar{x}}$ expresses the point estimation of parameter x,

### Intermediate clinical outcomes

The intermediate consequences measured were:

a. Pain: All patients were assessed for pain by a registered doctor immediately prior to randomization. A visual analogue scale from 1 to 10 was used: 0 = no pain; 1-2 = slight pain; 3-4 = mild pain; 5 = moderate pain; 6-9 = moderately severe pain; 10 = severe pain [11]. Pain scores were recorded twice daily by the doctors. Pain medication was given upon patient demand.

b. Clinical evaluation of the appearance of burn limits

A clinical evaluation of the appearance of burn limits was made each day by the doctor. We used a binary and a continuous variable to quantify these criteria. The modalities of the first variable were: 1. Healthy appearance of burn limits. 2. No Healthy appearance (presenting Redness, Swelling, Other). The choice of a binary variable was due to the lack of instruments that aggregated the extent of trouble caused by each type of no healthy appearance. The other variable was the time of no healthy appearance during the follow up.

c. Percentage of complications. We compared the percentage of complications that appeared during the treatment, (allergy and infection), in total groups and subgroups.

After discharge from the hospital the observation of patients concerning the pain and the burn limits was achieved through consultation and by the doctor's personal contact on the telephone.

### Statistical issues

#### Data imperfections

No patients refused to participate in the study. As referred to above, three patients were excluded of the study because of eligibility errors (un-reordered data). These eligibility errors did not create bias because the eligibility criteria of the study were defined objectively before treatment allocation [26]. There weren't censored observations for the final outcomes. We did have loss of contact for the pain measurement (9th day and after) for 3 patients recovered earlier than 8th day (1 for the MEBO group and 2 for the old therapy group). These censored observations were imputed by the Method of Last Observation Carried Forward, with decreased risk of bias because the censoring occurred near the end of the follow-up period [26].

#### Protocol induced costs and outcomes

Although the design of this study was pragmatically oriented, protocol induced outcomes were observed in the case of superficial partial MEBO group. Prolongation of in- hospital stay by 4-5 days was observed in the beginning of the study (despite the suggestions of the blinded evaluator), for six patients in order to measure TEWL indicator (as already mentioned, doctors utilized TEWL indicator measurement habitually on 1^st^, 4^th^, and 7^th ^day). These patients presented spontaneous re-epithelialization in very few days (2-3) days. This protocol induced costs and outcomes were easily detected and omitted by the final calculations [29].

#### Sample size

The sample size was determined during the 1rst year of the study as follows: The primary outcomes on which this study was powered were hospitalization days and the time of 50% wound healing. We had undertaken a pilot work to estimate effect sizes. It is known that, pilot studies offer many advantages in the estimation of an informed effect size target for power calculations [30]. The pilot work was carried out on 50 subjects. These 50 patients were the first (randomized) patients included in the study, so the study started, and then during the process it was decided on how many patients were still needed.

According to this work, MEBO substantially reduced the in hospital stay duration by 1.7 days in average (sd: 2.7). That is, with a 2-sided test of 5% the study has more than 90% power to detect a significant difference in hospitalization duration, given a sample size of 100 in each group.

Additionally, MEBO substantially reduced the time of 50% wound healing in the case of superficial partial thickness burns (n = 30) by 2.9 days in relation to the standard therapy (sd: 3.4) That is, with a 2-sided test of 5% the study has more than 90% power to detect a significant difference in time of wound healing, given a sample size of 50 in each group.

### Statistical Analyses

#### Patient characteristics, costs and outcome variables

Statistical tests of homogeneity for the different patient characteristics (e.g. gender, age, type of burns, skin photo type, burn surface area, TEWL at the 1rst day, toxic habits, co-morbidity etc), as well as for intermediate outcomes were used (χ^2 ^test variables t-test and Mann Whitney test for non normal data). All analyses were made according to the intention to treat principle.

#### Incremental Cost Effectiveness Ratio

Fieller's method was used to construct confidence intervals for the final outcomes in the case of total groups and superficial partial thickness burns groups. This method takes into account the potential asymmetry of the estimator of the ratio [31-34]. In the majority of existing research, it presented better performances than the other comparable methods and produced reasonably accurate confidence intervals [29].

Fieller's theorem is based on asymptotic normality theory:

$${\bar{\mu }}_{\Delta \text{C}}-\left(\text{ICER}\right){\bar{\mu }}_{\Delta \text{E}}\;\sim \text{N}\left(0,\text{1}\right)$$

Where $\bar{\mu_{\Delta \text{C}}}$ and $\bar{\mu_{\Delta \text{E}}}$ are respectively the means of Δ_μC _and Δ_μE_.

Fiellers confidence intervals can be calculated as:

$$(\bar{\bar{ICER}})\frac{1-{\text{z}}_{\alpha /2}^{2}\rho \text{cv}(\Delta_{\mu \text{C}})\text{cv}(\Delta_{\mu \text{E}})}{1-{\text{z}}_{\alpha .2}^{2}\text{cv}{\left(\Delta_{\mu \text{E}}\right)}^{2}}\pm (\bar{\bar{ICER}})*$$

$$*\frac{{\text{z}}_{\alpha /2}\sqrt{\text{cv}{\left(\Delta_{\mu C}\right)}^{2}+\text{cv}{\left(\Delta_{\mu \text{E}}\right)}^{2}-2\rho \text{cv}(\Delta_{\mu \text{C}})\text{cv}(\Delta_{\mu \text{E}})-{\text{z}}_{\alpha /2}^{2}(1-\rho^{2})\text{cv}{\left(\Delta_{\mu \text{C}}\right)}^{2}cv{\left(\Delta_{\mu \text{E}}\right)}^{2}}}{1-{\text{z}}_{\alpha /2}^{2}\text{cv}{\left(\Delta_{\mu \text{E}}\right)}^{2}}$$

Where ρ is the correlation coefficient for cost and effect differences and cv respectively the coefficient of variation.

**Figure 2:**
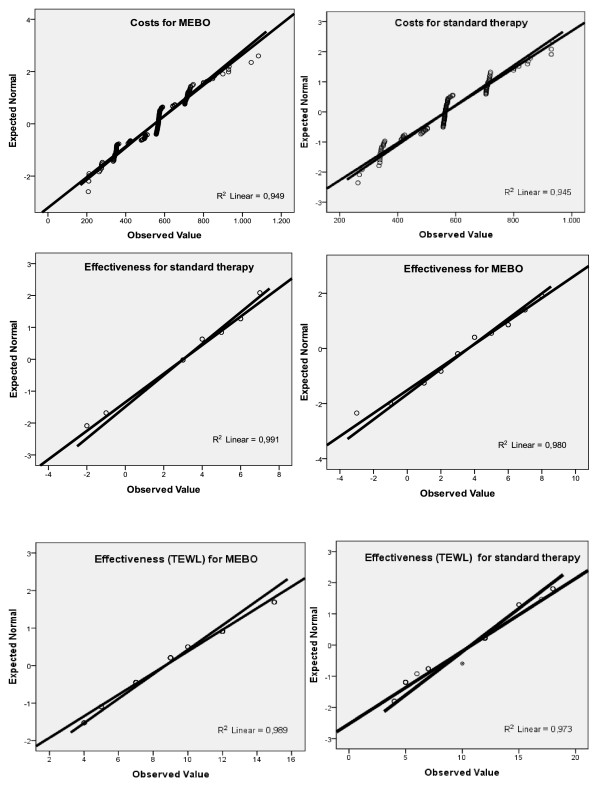
**Q-Q plots of costs and effectiveness for MEBO group and standard therapy group**.

**Figure 3:**
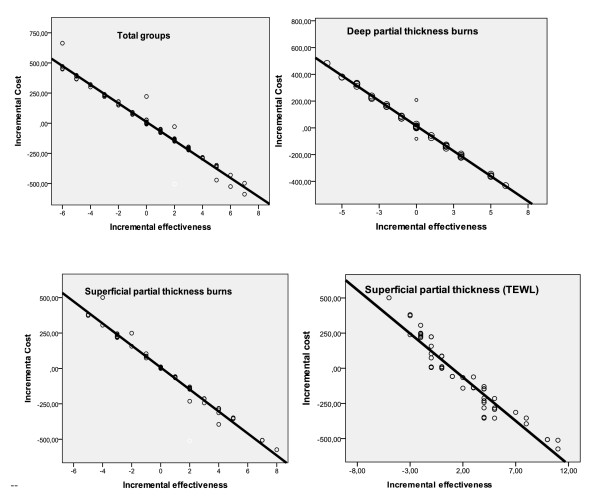
**Scatter plots of Incremental costs and effectiveness for total groups, groups with deep partial thickness burs and groups with superficial partial thickness burns**.

The normality assumption of the sample means of cost and effectiveness seemed to be reasonable in our case. As can be seen by the relative normal quantile plots (Q-Q) in Figure 2 where each observed value was plotted against the expected value from the normal distribution, the points in all cases clustered approximately around a straight line. Also, scatter-plots in Figure 3 gave us clear indication that cost and effect differences related to a linear function in all groups.

The acceptability criterion was used in the case of deep partial thickness burns group since the ICER denominator in this case wasn't statistically significant [35]. Cost Effectiveness Acceptability Curves (CEAC's), measured the probability that the CE ratio-resulting from a trial was acceptable in relation to different values of the ceiling ratio (from 0 to ∞).

The cost effectiveness acceptability curve was constructed by calculating the proportion of 2,500 bootstrap replications [35]. All analyses were conducted in SPSS and Excel Professional 2003.

## Results

### Patient's characteristics

Table 3 presents demographic and medical characteristics of the sample. Statistical tests of homogeneity for the different patient characteristics (e.g. gender, age, type of burns, photo-type, burn surface area, TEWL at the 1st day, toxic habits etc), as well as for intermediate outcomes were used (χ^2 ^test, t-test and Mann Whitney test for non normal data).

**Table 3:** Patient's characteristics and homogeneity tests

	MEBO GROUP			STANDARD THERAPY GROUP					
	**Total group**	**Deep partial thickness**	**Superficial thickness**	**Total groups**	**Deep partial thickness**	**Superficial thickness**	**Total groups**	**Deep partial thickness**	**Superficial thickness**

Patients	104	50	54	107	52	55			
Gender	Men: 60 Women: 44	Men: 30 Women: 20	Men: 30 Women: 24	Men: 71 Women: 36	Men: 30 Women: 22	Men: 41 Women: 14	χ^2 ^= 3.012 p = 0.08	χ^2 ^= 0.126 p = 0.722	χ^2 ^= 0.423 p: 0.04
Age (sd)	Mean: 42.62 (13.32)	Mean: 40.32 (13.3)	Mean: 44.74 (13.92)	Mean: 42.74 (14.58)	Mean: 43.94 (15.16)	Mean: 41.35 (13.08)	t-test -0.06 p = 0.949	t-test -1.28 p = 0.204	t-test -1,33 p = 0.184
Age > 60	15	7	8	16	9	7	Ζ = 0.012 p = 0.49	Ζ = 0.211 p = 0.42	Ζ = 0.100 p = 0.46

Photo-type	I: 1 II: 17 III: 69 IV: 17	Ι: 1 II: 8 III: 35 IV: 6	I: 0 II: 9 III: 34 IV: 11	I: 2 II: 21 III: 71 IV: 13	I: 2 II: 11 III: 34 IV: 5	I: 0 II: 9 III: 38 IV: 8	χ^2 ^= 1.27 p = 0.740	χ^2 ^= 0.687 p = 0.744	χ^2 ^= 1.24 p = 0.761
Toxic habitudes	Smoking: 30 Alcohol 10 Both 3	Smoking: 18 Alcohol 4 Both 1	Smoking: 12 Alcohol 4 Both 2	Smoking: 40 Alcohol 4 Both 1	Smoking: 25 Alcohol 0 Both 0	Smoking: 15 Alcohol 4 Both 1	Ζ = 0.47 p > 0.05	Ζ = 1.01 p > 0.05	Ζ = 0.98 p > 0.05
Type of burns	Flame: 56 Scald: 48	Flame: 27 Scald: 23	Flame31 Scald: 23	Flame: 57 Scald: 50	Flame: 26 Scald: 26	Flame31 Scald 24	χ^2 ^= 1.075 p = 0.576	χ^2 ^= 1.24 p = 0.761	χ^2 ^= 2.22 p = 0.37
TEWL at the 1st day	Median: 59 IQR: 27	Median: 75 IQR: 12. 5	Median: 48 IQR: 12.25	Median: 51 IQR: 15	Median: 69 IQR: 17	Median: 51 IQR: 4	U = 5,387 p = 0.688	U = 859 p = 0.003	U = 1,.250 p: 0.135
Allergies	Penicillin: 2 Grass: 3 Food 2	Penicillin: 2 Grass1 Food: 0	- Grass: 2 Food: 2	Unknown: 4 Grass: 1 Food: 3	Unknown: 2 Grass: 1 Food: 1	Unknown: 2 Food: 2	Z = 0.5 p > 0.05	Z = 0.96 p > 0.05	Z = 0.85 p > 0.05
	
Burn Surface Area (sd)	Mean: 10.26 (4.37) Range: 5-15	Mean: 9.74 (4.84) Range: 5-15	Mean: 10.74 (3.87) Median: 10 Range: 5-15	Mean: 9.89 (4.89) Range: 5-15	Mean: 10.04 (4.59) Range: 5-15	Mean: 9.75 (3.87) Median: 10 Range: 5-15	t = 0.53 p = 0.59	t = -0.4 p = 0.70	U: = 1,242.5 p = 0.70
Nationality	Greek: 75 Third World: 12 Ex-eastern: 17	Greek: 38 Third World: 5 Ex-eastern: 7	Greek: 37 Third World: 7 Ex-eastern: 10	Greek: 69 Third World: 23 Ex-eastern: 15	Greek: 35 Third World: 8 Ex-eastern: 9	Greek: 34 Third World: 15 Ex-eastern: 6	χ^2 ^= 4.73 p = 0.09	χ^2 ^= 1.48 p = 0.476	χ^2 ^= 4.02 p = 0.134

Patients of all groups had similar characteristics concerning the most important parameters such as burn size (p > 0.5 in all cases) and age (p > 0.5 in all cases). Exceptions were the cases of gender where the homogeneity was rejected (p = 0.04) for the superficial partial thickness burns groups.

### Final outcomes: gain in days of in-hospital stay, days needed for 50% wound healing

Significant differences were observed between MEBO and the standard therapy (p = 0.03) in effectiveness in total groups and in superficial partial thickness burn groups. MEBO further reduced by nearly 1 day (sd: 4.5) in average the in-hospital stay in relation to the standard therapy. This means it reduced the hospitalization time by nearly 20.6% (mean gain in days: 3.63, sd: 2.19 versus mean: 3.01, sd: 2.02). We have to underline at this point that this difference concerned moderate burn wounds (TBSA < 15%) needing conservative therapy that implies or otherwise dictates short times of hospitalization (≤ 10 days). So, the clinical significance of this find had to be evaluated by taking into account these special circumstances [27]. Also, MEBO was more effective than the standard therapy in the case of superficial partial burn wounds since: 1. It nearly reduced by 1 day (sd: 5.05) in average the in- hospital stay, that is it significantly reduced (p = 0.02) the hospitalization time by 29.63% in relation to the standard therapy (mean gain in days: 4.20, sd:2.1 versus mean:3.24, sd: 2.1). 2. It significantly reduced (p = 0.00) the time of 50% wound healing by 2 days in average in relation to the standard therapy group. That is, the time of 50% wound healing was shorter by 19.07% in relation to the standard therapy group (8.7 days, sd: 3.0 versus 10.75, sd: 3.8). On the contrary, in the case of deep partial thickness burns the differences observed in favor of MEBO group (mean gain in hospitalization stay: 3.02 days sd 2.1 versus 2.79 days sd 1.9) were not statistically significant. (p = 0.56).

### Cost results

Table 4 gives an overview of the mean costs for each group. For each group, we concluded the following:

**Table 4:** Mean costs, sd and incremental costs (in 2006 €)

	MEBO			STANDARD THERAPY			Differences and sd		
**Mean costs and sd**	**Total groups**	**Deep partial thickness burns**	**Superficial partial thickness burns**	**Total groups**	**Deep partial thickness burns**	**Superficial partial thickness burns**	**Total groups**	**Deep partial thickness burns**	**Superficial partial thickness burns**

Hospitalisation	463.5 163.35	512.12 155.10	418.48 159.11	512.22 148.45	529.11 141.12	496.25 154.65	-48.72 155.63	-16.99 146.7	-77.77 158.17
Antibiotics	33.91 29.70	34.29 29.43	33.56 30.23	31.41 16.42	29.93 12.8	32.80 19.25	2.5 23.84	4.36 22.3	0.76 25.5
Laboratory tests	0.70 2.53	0.94 2.92	0.48 2.1	0.63 2.45	0.80 2.81	0.47 2.08	0.07 2.48	0.14 2.84	0.01 2.1
Anti-inflammatory/anti-histaminic	0.61 1.92	0.92 2.33	0.33 1.41	0.00	0.00	0.00	0.61 1.35	0.92 1.61	0.33 1.00
Scrub products	2.24 1.01	2.38 1.01	2.12 1.01	2.46 1.06	2.47 0.95	2.44 1.17	-0.22 1.04	-0.09 0.97	-0.32 1.10
Visits	5.31 2.06	6.42 1.92	4.28 1.61	5.96 1.85	6.27 1.19	5.67 2.28	-0.65 1.95	0.15 1.57	-1.39 1.99
Analgesics	0.09 0.07	0.08 0.06	0.10 0.07	0.16 0.09	0.13 0.07	0.18 0.09	-0.07 0.07	-0.05 0.06	-0.08 0.08
Local agents	20.96 2.96	21.40 3.50	20.55 2.31	11.40 3.31	12.28 3.14	10.63 3.32	9.56 3.14	9.12 3.30	9.92 2.89
Mean time spent by doctors and nurses per course treatment	15.66 (2.98)	16.10 (2.86)	15.26 (3.05)	14.91 (2.93)	15.61 (2.79)	14.25 (2.93)	0.75 (5.43)	0.49 (2.4)	1.01 (5.26)
Total costs*	529.66 172.75	579.83 157.70	483.21 174.44	566.21 151.45	582.15 142.28	551.13 159.47	-36.55 161.94	-2.32 148.6	-67.92. 168.42

* Mean time spent by doctors and nurses per course of treatment not included. Numbers may not add up due to rounding.

1. Total groups: MEBO presented lower total cost per patient (although not significantly different: p = 0.10). The mean cost per patient was nearly by 36.55 € lower in the MEBO group. Significant differences in favor of the MEBO group were observed in the case of the mean cost of medical visits per patient (€5.31 versus €5.96, p = 0.01), hospitalization costs (€463.5 versus €512.22, p = 0.02) and costs of analgesics (€0.09 versus €016, p = 0.00). On the contrary, the mean cost of inflammatory medicines consumed by the MEBO group was significantly different from 0 (p = 0.00) and the mean cost of local agents was significantly higher in the MEBO group (€20.96 versus €11.40, p = 0.00). All other differences in partial costs were not significant. We have to mention also that the MEBO group presented an observed costlier consumption of antibiotics (mean: 33.91 versus mean: 31.41) not proved, nevertheless statistically significant (p = 0.09).

2. Deep partial thickness groups: Although the mean cost per patient was lower in the MEBO group these differences were not statistically significant (€579.83 versus €582.15, p = 0.938). The mean cost of analgesics was higher in the standard therapy group and this difference was statistically significant (€0.08 versus €0.13, p = 0.002). On the other hand, the cost of inflammatory medicines consumed by the MEBO group was significantly different from 0 (2.68 p = 0.00) and the cost of local agents was significantly higher in the MEBO group (€21.40 versus €12.28, p = 0.0). All other differences in mean partial costs were not statistically significant.

3. Superficial partial thickness groups: The total mean cost was significantly lower in the MEBO group (483.21 versus 551.13, p = 0.00). That is, the mean cost per patient was nearly by €67.92 lower in the MEBO group than in the standard therapy group. Highly significant differences in favor of MEBO group were observed in the case of medical visits (€4.28 versus €5.67, p = 0.00) hospitalization costs (418.48 versus 496.25, p = 0.01) and costs of analgesics (€0.10 versus €0.18 p = 0.00). On the contrary, the MEBO group received a quantity of inflammatory agents significantly different from 0 (p = 0.00) and the cost of local agents was significantly higher in the MEBO group (€20.55 versus €10.63, p = 0.00). All other differences observed in mean partial costs were not significant.

No significant differences were observed (p > 0.05 in all cases) between groups and subgroups on the time spent per course of treatment by doctors and nurses.

### Incremental cost effectiveness

The results are as follows:

1. Total groups. The corresponding ICER is €-58.95 per day of hospitalization gained which has a Fieller's confidence interval (CI) of (-63.10;-55.09) that indicates MEBO was more cost effective therapy than the standard therapy. Nevertheless these results must be interpreted with caution because of the non significance of the numerator.

**Figure 4:**
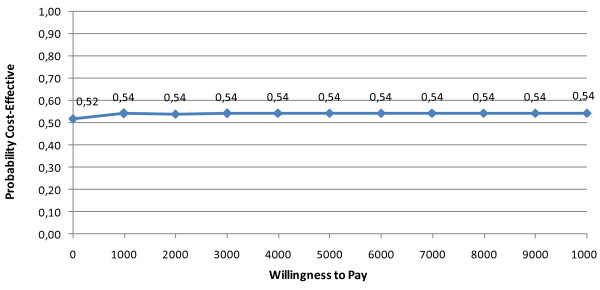
**Cost effectiveness acceptability curve MEBO vs. standard therapy (groups with deep partial thickness burns)**.

Deep partial thickness burns group. The corresponding ICER was €-10.1 per day of hospitalization gained which had a 95% bootstrap confidence interval, (based on the 2.5th and 97.5th percentile of the distribution of the 2, 500 bootstrapped ICERs), of (-120.4; 181.8), indicating a large amount of stochastic uncertainty. The acceptability curve (Figure 4) cut the y-axis at 0.51 because only 51% of the density involved cost savings and asymptotes to 0.54 because 54% of the density involved health gains by the use of the MEBO therapy [34]. As can be seen, beyond € 1,000 the acceptability curve became flat and was then fairly insensitive to Willingness to pay (WTP) levels. This indicated that a decision-maker's choice at WTP € 1,000 and over was probably not surrounded by much uncertainty, even when the ICERs had broad confidence intervals.

2. Superficial partial thickness burns group. MEBO was the dominant therapy under the point of view of the gain in hospitalization stay (ICER: €-70.75 per day of hospitalization gained, 95% CI:-74.24;-67.58), as well as under the point of view of TEWL (mean ICER: €-33.16 per day of recovery gained: 95%CI: -34.49;-31.89). MEBO presented in both cases superior effectiveness at a lower total cost.

### Intermediate outcome results: pain, complications, no healthy appearance of burn limits

A greater proportion of patients in the standard therapy group received paracetamol (95.3% versus 84.3%) as well as paracetamol+codeine (48.6% versus 30.77%). Also, the standard therapy group in total groups and in superficial partial thickness group received a greater quantity of paracetamol (total groups: median: 3,750, IQR:5300, superficial partial thickness group (median: 4,750, IQR:3,200) than the MEBO therapy group (total groups: median:1500, IQR:1463,5, superficial partial thickness group: median:2,500, IQR:2,250). These differences were both highly significant (p = 0.00). Also, the standard therapy group in total groups and in superficial partial thickness group received a significantly greater (p = 0.00) quantity of paracetamol+ codeine (total groups: median:2,700 IQR:1,463, superficial partial thickness group: median:2,700, IQR: 1,687) than the MEBO group (total groups: median:1125, IQR:900, superficial partial thickness group: median:900, IQR:2,250).

In the case of deep partial thickness burns highly significant differences were observed in the case of paracetamol with the standard therapy group presenting a higher consumption of paracetamol (median:2,750, IQR:1,687) than the MEBO group (median:900, IQR:2,250). Nevertheless, no significant differences were observed in the consumption of paracetamol + codeine between these groups (p = 0.05).

**Figure 5:**
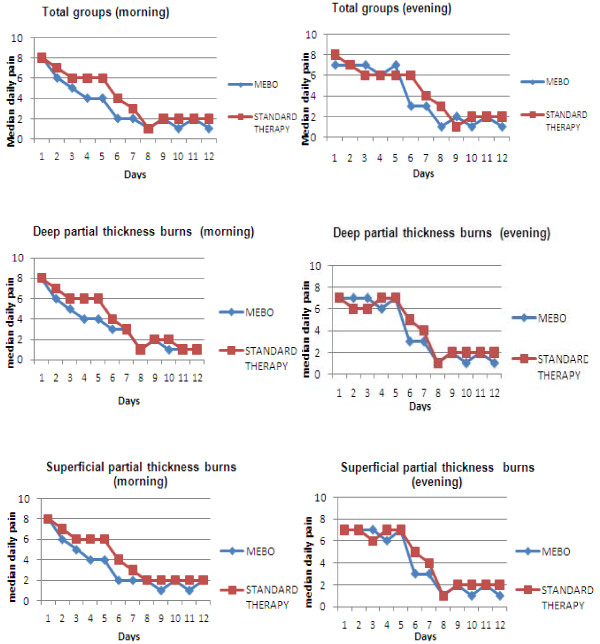
**Median daily pain (morning and evening) for total groups, groups with deep partial thickness burs and groups with superficial partial thickness burns**.

Figure 5 shows the median pain profiles for the morning, afternoon and evening assessments respectively, for the first 12 days. In the first days of therapy, the profile was more similar for the 2 treatment groups, whereas the median pain scores are significantly lower for MEBO (p = 0.00) after approximately the 2^nd ^post burn day in the morning or the 3^rd ^day in the evening with no major differences between the treatment groups after the 8^th ^post burn day.

**Figure 6:**
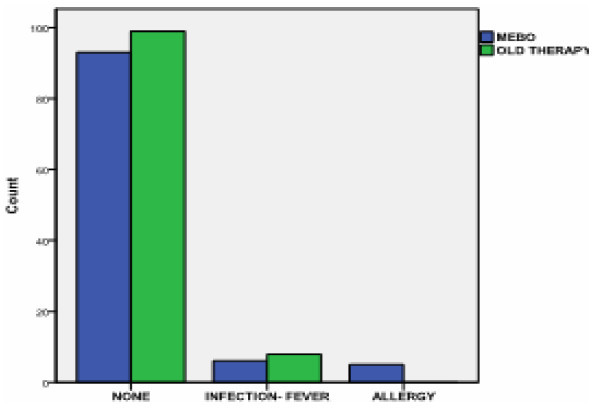
**Distribution of total groups by complication appearance**.

11 out of 104 patients in MEBO group versus 8 out 107 patients in the standard therapy group presented complications (Figure 6). These differences aren't statistically significant (p = 0.07). Five patients on the MEBO group (3 from deep partial thickness burns group and 2 from superficial partial thickness burns group) presented mild allergic reaction. During the period of the allergy Dimethindene cream was prescribed to these patients for 6 το 7 days whereas one patient received additionally methylprednisonole.

The incidence of wound infection (Staphylococcus, Pseudomonas) was similar (p = 0.62) in the 2 groups. Pseudomonas was more frequently presented in MEBO patients (4 out of 6 patients) than in the standard therapy group (3 out of 8 patients), although Staphylococcus wound infection was more frequent in the standard therapy group (5 out of 8 patients versus 2 out of 6 patients in the MEBO group). We have to note that the higher prevalence of Pseudomonas pathogen in the MEBO group can explain the observed higher costs (although not significantly different) in antibiotics consumption in this group in relation to the standard therapy group.

38 out of 104 patents and 28 out of 107 presented no healthy appearance of burn limits for some days (Figure 6). These differences aren't significant (p = 0.10). In the case of deep partial thickness burns, 20 out of 50 for MEBO group versus 13 out of 52 for the standard therapy presented no healthy appearance of burn limits for some days, but these differences are not significant (p = 0.10). Finally, in the case a of the superficial partial group 18 out of 54 in the MEBO group versus 15 out of 55 in the standard therapy group presented no healthy appearance of burn limits for some days although these differences aren't statistically significant (p = 0.49).

**Figure 7:**
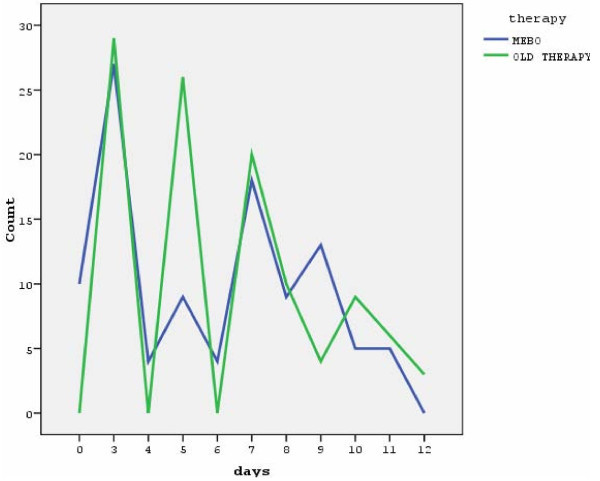
**Time of no healthy appearance of burn limits (total groups)**.

The median time of no healthy appearance, (Figure 7), was shorter in the standard therapy group (total groups), that is 5 days (IQR:5) versus 6 days (IQR: 5) for the MEBO group although these differences are not statistically significant (p = 0.29). No significant differences were observed in the median time of no healthy appearance of the burn limits in deep partial thickness burn groups (p = 0.44). Nevertheless, this time was shorter in the standard therapy group (median: 5.00, IQR:5) than in the MEBO group, (median:7.00, IQR: 4). On the contrary, in the groups with superficial-partial thickness burns the median time of no healthy appearance was significantly shorter in the MEBO group (median:4.00, IQR:5.25) than in the standard therapy group (median:5.00, IQR:3) and this difference was statistically significant (p = 0.02).

### Sensitivity analysis

**Figure 8:**
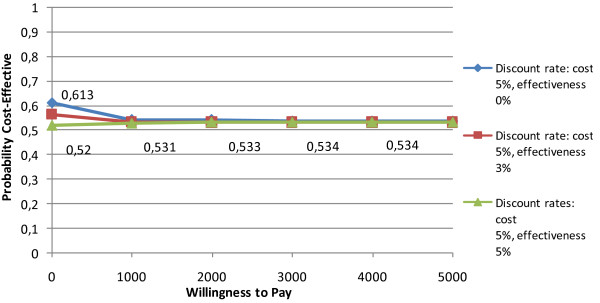
**Cost effectiveness acceptability curves for deep partial thickness burns (discount rates: 0%,3%, 5%)**.

The above analyses were repeated for the hypothetical scenarios of discounting costs and outcomes by 3% and 5% because of the unfavourable economic situation in the country and the increased deficiencies in nursing personnel. Table 5 summarizes the results of sensitivity analysis. As can be seen, the analyses did not reverse the findings. Figure 8 presents the cost effectiveness acceptability curves corresponding to three different discount rates for effectiveness in the case of deep partial thickness burn groups. As can be seen, beyond the WTP ceiling of €1,000 the acceptability continued to be fairly insensitive to WTP levels. 57% up to 61% of the joint density involves monetary gains and 53% up to 54% of the joint density involves health gains by the use of MEBO, under the different scenarios described in Figure 8. Nevertheless, we have to underline that uncertainty remains particularly important in this case and these indications should be interpreted with caution.

**Table 5:** Results of sensitivity analysis (Total groups and superficial partial thickness burns)

Discount rate	Groups	ICER and Fieller's CI	
		
		Gain in days of hospitalisation	Gain in days of recovery (TEWL)
3%	Total groups	-60.72 (CI:-64.92;-56.76) to -57.23 (CI-53.48;-61.19)	_
3%	Groups with superficial partial thickness burns	-73,04(CI-78.14;-68.55) to -68.70 (CI-73.34;-64.58)	-34.13 (CI:-35.07;-33.21) to -32.17 (CI:-33.51;-30.93)
5%	Total groups	-61.95 (CI:-66.27;-57.88) to -56.14 CI:(-60.67;-52.41)	_
5%	Groups with superficial partial thickness burns	-74.64 (-79.97;-69.96) to -67.38(-71.93;-63.34)	-34.83 (CI:-36.24;-33.46) to -31,56(CI:-32.43;-30.48)

## Discussion

In the total groups MEBO presented lower cost (although not significantly different: p = 0.10) and better effectiveness. That is, this randomized trial supported the hypothesis that MEBO reduces the time of hospitalization. The effectiveness results were consistent with the results of previous studies [10-16]. Also, the data suggest that *MEBO *was the dominant therapy for superficial partial thickness burns with significantly lower costs and significantly superior effectiveness due to a lesser time of recovery. In this case the results support the corresponding hypotheses i and ii that MEBO reduces the time of hospitalization and wound healing as well as the costs of recovery. On the contrary, no significant differences were observed in cost and effectiveness in the case of deep partial thickness burns group, so in this case the data do not support the above mentioned tested hypotheses. *Also, results in all groups suggest that MEBO *reduces pain levels and presents similar percentages of complications with the comparator, supporting the corresponding hypotheses iii and iv of this study.

Some limitations of this study were found to merit discussion:

The major limitation of this study is that it was single centred. Nevertheless, the setting was representative of the usual care in Greece and the sample was representative of the patients hospitalized for burns. (The annual burn sample of the specific area in our study exceeded 10% of the total potential eligible population, a fact that may assure the external validity of the study).

Second, the statistical power was inadequate (1-β = 0. 14) in the case of deep partial thickness burn groups to detect significant differences. Nevertheless, it did not threaten the external -validity of the study, which was designed for the total number of patients with TBSA < 15%.

Third, the follow-up of this study was rather short (18 days). Nevertheless, other studies concurred with our results and found that scar quality was superior in wounds treated with MEBO [10,11,15,16].

Fourth, although the design of this study was pragmatically oriented, protocol induced costs and outcomes were observed. These protocol induced costs and outcomes were easily detected and were omitted. Nevertheless, as protocol induced costs and outcomes have appeared it may be difficult or impossible to exclude the full impact of these services of the analysis and this could bias the final differences [36]. On the other hand, in-hospital stay constitutes a more pragmatic but also subjective measure of effectiveness. Also, the research was not blinded because *Povidone Iodine *has a characteristic odour and colour and was therefore recog-nizable. An unblinding trial could be highly susceptible to classification bias [26,35]. Nevertheless, the persons evaluating treatment outcomes were blinded to treatment group assignment in order to eliminate classification bias [37].

Fifth, there was a general problem in the cost effectiveness studies of local agents because of the inconclusive evidence regarding the efficacy of local agents on burn wounds [37]. A trial based evaluation such as the described could lead to erroneous conclusions for it may prove a therapy as cost effective simply because the comparator is completely ineffective. In this study this inconclusive evidence can be accelerated by the fact that the comparator was a known cytotoxic agent [38] with deleterious effects in wound to the keratinocytes and fibroblasts. Nevertheless in Greece up to this day *Povidone Iodine *is the first line treatment modality for burns of this kind essentially because of its antimicrobial effects [39].

Finally, the sensitivity analysis compared the actual situation with some hypothetical scenarios that were somewhat arbitrary, and the trade-offs between costs and effects may have been different from what was presented. Appearance of allergy on some patients of the MEBO group as well as the higher prevalence in this group of Pseudomonas pathogens needs also further exploration.

## Conclusions

Results suggest that topical application of *MEBO *may be considered for further investigation as a potential first-line treatment modality for superficial partial thickness burns. *MEBO*, in this case, presents similar percentages of complications with the comparator, lower pain levels and shorter time of no healthy appearance of the burn limits. Also, it constitutes the dominating therapy based on cost effectiveness aggregation. Nevertheless, in light of the above mentioned limitations our findings should be interpreted with some caution and must be verified in a larger multi-center trial. It is our recommendation that such a trial should be conducted in the near future.

## List of abbreviations

CI: Confidence intervals; ICER: Incremental Cost Effectiveness Ratio; IQR: Interquartile range; MEBO: *Moist Exposed Burn Ointment, Povidone iodine plus bepanthenol cream: standard therapy*; sd: Standard deviation, TBSA: Total Body Surface Area; TEWL: Transepidermal Water Loss

## Competing interests

The authors declare that they have no competing interests.

## Authors' contributions

VJC has participated on the design, has written the text, and made the statistical analyses, EGT has participated on the design and in the data collection GCHS has participated on the design and data collection, FNA has participated in the data collection. JDI was the instigator of the concept and has contributed to the methodology of this study. Αll authors read and approved the final manuscript.

## Pre-publication history

The pre-publication history for this paper can be accessed here:

http://www.biomedcentral.com/1472-6882/11/122/prepub
